# Supplementary material for: Geraniol-Mediated Suppression of Endoplasmic Reticulum Stress Protects against Cerebral Ischemia–Reperfusion Injury via the PERK-ATF4-CHOP Pathway
Source: Int J Mol Sci. 2022 Dec 29;24(1):544. doi: 10.3390/ijms24010544 (PMC9820715; doi:10.3390/ijms24010544)
Supplement: Supplementary file 1 [file ijms-24-00544-s001.zip › ijms-1874931-supplementary.pdf]

Table S1: Summary of 59 recognized ER stress -associated genes.

| **Gene** | **Type** | **Source** |
|---|---|---|
| Eif2a | Endoplasmic reticulum stress | Li HQ, Xia SN, Xu SY, Liu PY, Gu Y, Bao XY, Xu Y, Cao X. γ-Glutamylcysteine Alleviates Ischemic Stroke-Induced Neuronal Apoptosis by Inhibiting ROS-Mediated Endoplasmic Reticulum Stress. Oxid Med Cell Longev. 2021 Nov 16;2021:2961079. doi: 10.1155/2021/2961079. PMID: 34824669; PMCID: PMC8610689. [39] |
| caspase 12 | Endoplasmic reticulum stress | |
| Hsp90aa1 | Endoplasmic reticulum stress | |
| Hspa1a | Endoplasmic reticulum stress | |
| Bax | Endoplasmic reticulum stress | |
| Xbp1 | Endoplasmic reticulum stress | |
| Eif2ak4 | Endoplasmic reticulum stress | biological information database<br><br>Liu S, Liu J, Wang Y, Deng L, Chen S, Wang X, Zuo T, Hu Q, Rao J, Wang Q, Dong Z. Differentially expressed genes induced by β-caryophyllene in a rat model of cerebral ischemia-reperfusion injury. Life Sci. 2021 May 15;273:119293. doi: 10.1016/j.lfs.2021.119293. Epub 2021 Mar 8. PMID: 33705733. [52] |
| Eif2ak2 | Endoplasmic reticulum stress | |
| Eif2ak3 | Endoplasmic reticulum stress | |
| Atf1 | Endoplasmic reticulum stress | |
| Atf6b | Endoplasmic reticulum stress | |
| caspase-3 | Endoplasmic reticulum stress | |
| caspase 6 | Endoplasmic reticulum stress | |
| caspase 9 | Endoplasmic reticulum stress | |
| caspase 14 | Endoplasmic reticulum stress | |
| caspase 7 | Endoplasmic reticulum stress | |
| caspase 8 | Endoplasmic reticulum stress | |
| caspase 4 | Endoplasmic reticulum stress | |
| Bcl6 | Endoplasmic reticulum | |

| | | |
|---|---|---|
| | stress | |
| Tmbim6 | Endoplasmic reticulum stress | |
| Hspa5 | Endoplasmic reticulum stress | |
| Prkra | Endoplasmic reticulum stress | |
| Creb3l1 | Endoplasmic reticulum stress | |
| Creb3l3 | Endoplasmic reticulum stress | |
| Creb3 | Endoplasmic reticulum stress | |
| Crebbp | Endoplasmic reticulum stress | |
| Creb3l2 | Endoplasmic reticulum stress | |
| Creb3l4 | Endoplasmic reticulum stress | |
| Ern2 | Endoplasmic reticulum stress | |
| Rnasel | Endoplasmic reticulum stress | |
| Eif2ak1 | Endoplasmic reticulum stress | |
| Crebzf | Endoplasmic reticulum stress | |
| Aatf | Endoplasmic reticulum stress | |
| caspase 2 | Endoplasmic reticulum stress | |
| caspase 1 | Endoplasmic reticulum stress | |
| Caap1 | Endoplasmic reticulum stress | |
| Bcl6b | Endoplasmic reticulum stress | |
| Bcl2a1 | Endoplasmic reticulum stress | |
| Bcl2l15 | Endoplasmic reticulum stress | |
| Bag1 | Endoplasmic reticulum stress | |

| | | |
|---|---|---|
| Bcl2l10 | Endoplasmic reticulum stress | |
| Bcl2l11 | Endoplasmic reticulum stress | |
| Bcl2l1 | Endoplasmic reticulum stress | |
| Bcl2l13 | Endoplasmic reticulum stress | |
| Bak1 | Endoplasmic reticulum stress | |
| Hsp90ab1 | Endoplasmic reticulum stress | |
| Hsp90b2 | Endoplasmic reticulum stress | |
| Rnasel | Endoplasmic reticulum stress | |
| Crebrf | Endoplasmic reticulum stress | |
| Bcl2l12 | Endoplasmic reticulum stress | |
| Bag5 | Endoplasmic reticulum stress | |
| Bag2 | Endoplasmic reticulum stress | |
| Bcl2l14 | Endoplasmic reticulum stress | |
